# Vitamin K alleviates bone calcium loss caused by *Salmonella* Enteritidis through carboxylation of osteocalcin

**DOI:** 10.1186/s40104-021-00604-z

**Published:** 2021-07-13

**Authors:** Yaojun Liu, Rainer Mosenthin, Lihong Zhao, Jianyun Zhang, Cheng Ji, Qiugang Ma

**Affiliations:** 1 State Key Laboratory of Animal Nutrition, College of Animal Science and Technology, China Agricultural University, Beijing, 100193 China; 2 Institute of Animal Science, University of Hohenheim, 70593 Stuttgart, Germany

**Keywords:** Calcium, Laying hen, *Salmonella* Enteritidis, Vitamin K

## Abstract

**Background:**

The present study aimed at evaluating the effect of vitamin K (VK) supplementation on bone health of laying hens challenged by *Salmonella* Enteritidis.

**Methods:**

A total of 80 32-week-old double negative *salmonella*-free brown-egg laying hens were randomly assigned to 4 treatments with 20 replicates each (1 bird per replicate) according to a 2 × 2 factorial design with 2 dietary VK supplementation levels [0 mg/kg (VK0) vs 2 mg/kg VK (VK2) and 2 challenge treatments [*Salmonella* Enteritidis (SE) vs physiological saline solution (PS)]. During the last 3 days of week 43 of age, birds of both VK treatments were either orally challenged with 1.0 mL suspension of 10^9^ cfu/mL *S.* Enteritidis daily or received the same volume of PS.

**Results:**

The laying rate, daily egg mass, tibia strength, CT, cOC and cOC/(cOC + ucOC) of VK2 treatment increased (*P* < 0.05) in contrast to VK0, however, the medullary area and ucOC of VK2 treatment decreased (*P* < 0.05) in contrast to VK0. Mortality, medullary area, serum Ca content of SE treatments increased (*P* < 0.05) in contrast to PS treatments. In both SE treatments, the decrease (*P* < 0.05) in birds’ tibia strength was associated with higher (*P* < 0.05) Ca levels in serum. There is an interaction (*P* < 0.05) between SE challenge and VK levels with regard to tibia strength and serum Ca levels. At week 42, serum CT was positively correlated with cOC (R = 0.99, *P* = 0.009); at week 44, tibia strength was positively correlated with BMD (R = 0.95, *P* = 0.045), but negatively correlated with medullary area (R = − 0.98, *P* = 0.018).

**Conclusions:**

VK (2 mg/kg) supplementation to diets of laying hens can enhance bone strength under challenge situations with *Salmonella* Enteritidis. Medullary area has proven to be a sensitive biomarker for bone calcium loss caused by SE infection.

## Background

Some *Salmonella* serogroups are significant pathogens responsible for bone infections [1, 2]. *Salmonella enterica* serovar Enteritidis (*Salmonella* Enteritidis), a Gram-negative bacterium, is the prevalent egg-product-related foodborne pathogen [3]. Infections with *Salmonella* Enteritidis (SE) are known to affect the health conditions of chickens [4, 5] resulting in the loss of homeostasis which, in turn, will reduce chickens’ production performance.

*Salmonella*, well-recognized as an intracellular pathogen, has the ability to invade into and persist within osteoblasts [6]. Bacterial infections and their products have been described as potent stimulators of osteoclastogenesis and bone resorption [7].

Efforts aiming to maintain birds’ health comprise the use of various dietary supplements [8, 9] including vitamin K (VK). Vitamin K is an essential fat-soluble micronutrient. Besides its function in the blood coagulation pathway, VK has also been considered as important factor in bone health [10]. Bone health is orchestrated by dynamic balance of minerals, organic matrices and hormones. The skeleton is the major reservoir for providing calcium. Many functions of Ca are regulated by calcitonin (CT), parathyroid hormone (PTH), osteocalcin (OC), carboxylated osteocalcin (cOC, the active type of osteocalcin), undercarboxylated osteocalcin (ucOC, the inactive type of osteocalcin) [11]. According to recommendations both of the National Research Council (1994, NRC) and the Chinese Feeding Standard of Chicken (Ministry of Agriculture of People’s Republic of China, 2004), VK should be supplemented at a level of 0.5 mg/kg to the diet of laying hens. However, under practical conditions, both feed industry and producer prefer to supplement diets for laying hens with up to 2 mg VK/kg to optimize laying performance and bone health [12]. There are no reports so far on the effect of VK on bone health of laying hens exposed to SE. Thus, the objective of this work was to study in birds orally challenged with SE, if dietary supplementation of VK may protect bones against SE-induced bone injury.

## Materials and methods

The experimental protocols were approved by the Animal Care and Use Committee of China Agricultural University (Beijing, China).

### Experimental design, feeding and housing management

Prior to challenge, cloacal swab samples and serum samples were taken from all birds, and were tested for the presence of SE (Poultry Microbiological Lab, China Agricultural University, Beijing, China). Birds devoid of *Salmonella,* based on these 2 tests, were referred to as “double negative”. Cloacal samples were pre-enriched with tetrathionate broth (CM 203–01, Land Bridge Technology Ltd., Beijing, China) at 37 °C for 24 h, and then streaked on Bismuth sulphite agar (CM 207, Land Bridge Technology Ltd.) [13]. Serum samples were tested in replicates for SE specific antibodies using an enzyme-linked immunosorbent assays (ELISA) (Shanghai Yuanmu Biotechnology Co., Ltd) according to the manufacturer’s instruction.

**Fig. 1:**
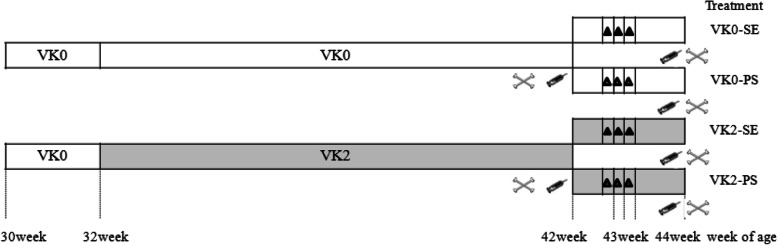
Experimental treatments and timeline of vitamin K supply and birds’ challenge.: blood sampling;: tibia sampling;: *Salmonella* Enteritidis (SE) challenge;: challenge physiological saline solution (PS);:dietary supplementation with 0 mg/kg vitamin K (VK0);:dietary supplementation with 2 mg/kg vitamin K (VK2)

In total, 80 32-week-old double negative brown-egg laying hens (Beijing Yukou Poultry Co., Ltd., China) with similar laying rate (93.0 ± 1.4%) and body weight (1.52 ± 0.12 kg) were randomly allotted to 4 treatments with 20 replicates each (1 bird per replicate) according to a 2 × 2 factorial design with 2 dietary VK (menaphtone sodium bisulphite (MSB), purity 50%; Yunnan Luliang peacechem Technology Co., Ltd.) supplementation levels [0 mg/kg (VK 0) vs. 2 mg/kg VK (VK 2)] and 2 challenge treatments [SE vs. physiological saline solution (PS)]. Layers were caged individually (wire floored) from week 30 to 44. The 4 treatments were as follows (Fig. 1): VK0-PS (basal diet + 0 mg/kg VK + PS), VK0-SE (basal diet + 0 mg/kg VK + SE), VK2-PS (basal diet + 2 mg/kg VK + PS), VK2-SE (basal diet + 2 mg/kg VK + SE). Challenged and non-challenged birds were kept in 2 independent, mechanically ventilated, hen houses under the same environmental conditions to avoid cross-infection. The lighting schedule was 16-h-light throughout the experiment. Room temperature was maintained at 23 ± 2 °C. All birds had free access to diets and water. The basal diet was a corn-soybean meal-based mash diet (Table 1), which was formulated to meet the Chinese Feeding Standard of Chickens (Ministry of Agriculture of People’s Republic of China, 2004), except for VK, which was added to the basal diet according to the experimental design. The concentrations of VK3 in the basal diet (VK0) was not detected. The concentrations of VK3 in treatment diet (VK2) was 2.3 mg/kg in according to the HPLC method. The whole experiment comprised 14 weeks (from 210 to 308 days of age) consisting of 2 weeks adaptation period and 12 weeks experimental period. Before and during the experiment, the *salmonella*-free status both of diets and water was determined by using PCR extract bacterial DNA [14].

**Table 1:** Ingredient composition and nutrient content of basal diet (%, DM)

Ingredient	%	Nutrient and energy^c^	
Corn	66.45	CP	15.52%
Soybean meal	22.80	AME	2,700, kcal/kg
Limestone	8.20	VK3	0 mg/kg
Dicalcium phosphate	1.70	Lys	0.75%
Sodium chloride	0.30	Met	0.37%
DL-Met	0.12	Met + Cys	0.64%
Choline chloride	0.10	Thr	0.57%
Vitamin premix^a^	0.30	Ca	3.60%
Mineral premix^b^	0.03	Total P	0.65%
Total	100	Available P	0.39%

^a^Vitamin premix supplied (per kg of diet): vitamin A, 6,000 IU; vitamin D_3_, 1,500 IU; vitamin E, 15 IU; vitamin B_1_, 3 mg; vitamin B_2_, 10.2 mg; folic acid, 0.9 mg; calcium pantothenate, 15 mg; niacin, 45 mg; vitamin B_6_, 5.4 mg; vitamin B_12_, 24 μg; biotin, 150 μg

^b^Mineral premix provided (per kg of diet): Cu (CuSO_4_·5H_2_O), 6.8 mg; Fe (FeSO_4_·7H_2_O), 66 mg; Zn (ZnSO_4_·7H_2_O), 83 mg; Mn (MnSO_4_·H_2_O), 80 mg; I (KI), 1 mg; Se (Na_2_SeO_3_), 0.3 mg

^c^Contents of VK, CP and Ca were analyzed. Contents of other nutrients and energy content were calculated based on Feeding Standard of Chickens (Ministry of Agriculture of People’s Republic of China, 2004)

### *Salmonella* Enteritidis inoculum and challenge

*Salmonella* *enterica* spp. *enterica* *serovar *Enteritidis (preservation number CVCC3377) was obtained from the China Institute of Veterinary Drug Control (Beijing, China). The frozen culture was recovered by using 10 mL of sterile tryptone soy broth and incubated at 37 °C with orbital shaking for 24 h. Subsequently, 5 mL of *S. enteritidis* pre-culture were transferred to 100 mL of tryptone soy broth and incubated with orbital shaking at 37 °C for 16–18 h. To determine the concentration of viable *S. enteritidis* in the culture, the inoculum was diluted with sterile phosphate buffer saline (PBS) (pH = 7.2), then plated on xylose lysine doxycholate (XLD) and incubated at 37 °C for 24 h. The stock culture was prepared in sterile PBS and adjusted to 1 × 10^9^ cfu/ mL of *S. enteritidis* to be used as inoculum [15]. During the last 3 days of week 43 of age, birds were orally challenged with 1.0 mL suspension of 10^9^ cfu/mL *S.* Enteritidis daily, whereas VK0-PS and VK2-PS treatments received the same volume of PS. A syringe with an attached flexible tube was used for the administration of the suspension and the physiological saline solution.

### Laying performance

Egg weight and egg production were recorded daily in replicates. Daily egg mass was recorded and expressed on hen per day basis. Feed consumption was measured every week. Broken eggs and bird mortality were recorded daily in replicates.

### Sample collection

Diets were analyzed for crude protein, calcium, total phosphorus and VK according to methods of Association of Official Analytical Chemists (AOAC, 1990). On the last day of week 44, 6 birds of each treatment were fasted for 12 h. Whole blood was collected via brachial vein, and kept in uncoated serum tubes at 12:00 h. Thereafter, serum was centrifuged at 1,000×*g* for 10 min at 4 °C and stored at − 20 °C.

Tibia was collected from left and right legs to determine fresh and dry tibia weight, tibia length, tibia breaking strength, bone mineral density, and bone Ca content.

### Tibia collection and analysis

**Fig. 2:**
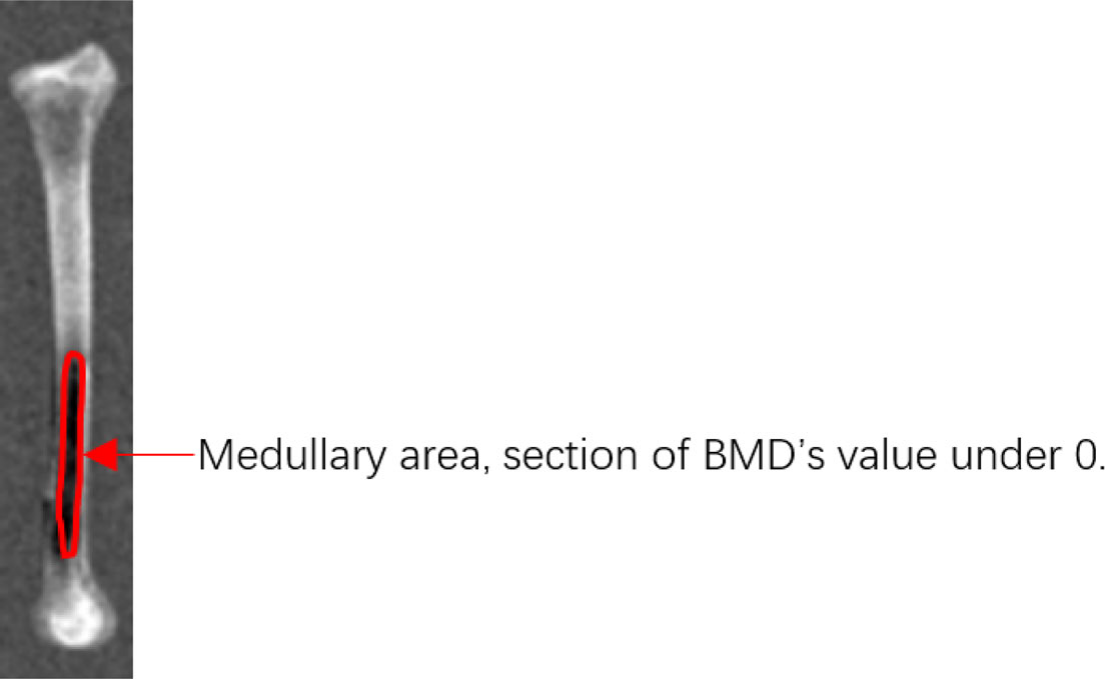
A photo illustrating the medullary area defined as section of BMD’s value under 0

Tibia samples of the left and right legs were imaged using XR-36 Automatic Scanner (Norland Medical System. Inc. USA) to determine tibia length, bone mineral density (BMD) and medullary area (the section of BMD’s value under 0 was considered as medullary area, Fig. 2). The weight of fresh samples of the whole tibia was determined before samples were dried in an oven at 100 °C for 24 h until constant weight. Tibia strength was measured by means of a three-point bending test using the MTS-810 Material Test System (MTS Ltd. USA). The right tibia was cleaned and then dried at 105 °C for 24 h. Thereafter, the tibia was defatted for 7 d by using ether, and then dried again at 100 °C for 24 h and weighed. Bone samples were ashed at 600 °C overnight in a muffle furnace [16]. Bone calcium content was determined by ethylenediamine tetraacetic acid titration method described by Manobhavan [17].

### Serum biochemical index

Serum Ca content was measured using a detection kit (Prodia diagnostics, Boetzingen, Germany) and a Hitachi 7600 automatic biochemical analyzer (Hitachi Co., Ltd., Tokyo, Japan). The serum CT, PTH, cOC, ucOC concentrations were determined by radioimmunoassay using commercial kits (Beijing North Institute of Biological Technology, Beijing, China). The ratio of cOC/(cOC + ucOC) was calculated to determine the percentage of active type of osteocalcin compared to the total of active and inactive types of osteocalcin.

### Statistical analysis

Data were analyzed using SAS statistical software (2010, version 9.2, SAS Institute Inc., Cary, NC, USA). Data obtained before SE challenge were analyzed by a one-way ANOVA using the General Linear Model (GLM) procedure, whereas data obtained after SE challenge were analyzed according to a two-way ANOVA for a 2 × 2 factorial design using GLM procedure. The main effects of VK supplementation levels and SE challenge status as well as their interactions were determined. Tukey’s multiple comparison test was used to separate means when interactive effects differed significantly. Data of broken eggs and birds’ mortality were transformed to arcsine values prior to statistical analysis. Results were expressed as treatment means with their standard error of the mean (SEM). A *P* value of < 0.05 was considered to be statistically significant. Pearson’s correlation coefficient analysis was conducted to determine correlations between variables. The individual correlations were visualized by using the ‘corrplot’ package in R software.

## Results

### Laying performance

During week 32 to 42, no difference (*P* > 0.05) was observed in laying performance between treatments (Table 2). During week 43 to 44, laying rate and egg mass were affected (*P* < 0.05) by the main effect of VK supplementation. Laying rate and egg mass in VK2 treatment were higher (*P* = 0.046 and *P* = 0.032, respectively) in comparison to VK0 treatments (91.6 vs 85.8 and 55.21 vs 51.56, respectively). Mortality was affected (*P* < 0.05) by the main effect of SE challenge as mortality in SE treatments increased (*P* < 0.010) from 18.4% to 26.6%.

**Table 2:** Effect of dietary vitamin K (VK) supplementation and *Salmonella* Enteritidis (SE) challenge on laying performance of laying hens (*n* = 20)

VK, mg/kg	SE^c^	Laying rate, %		Daily egg mass, g		Egg weight, g	Daily feed intake, g/d/bird			Feed conversion, g/g		Broken eggs, %		Mortality, %	
		Week 32–42	Week 43–44	Week 32–42	Week 43–44	Week 32–42	Week 43–44	Week 32–42	Week 43–44	Week 32–42	Week 43–44	Week 32–42	Week 43–44	Week 32–42	Week 43–44
0	–	92.6	87.1	55.24	52.49	59.62	60.23	116.91	101.20	2.11	1.93	3.16	0.00	11.9	18.4
0	+		84.5		50.64		59.99		99.32		1.96		0.00		26.6
2	–	94.5	92.4	56.75	55.92	60.06	60.49	114.83	105.23	2.03	1.89	2.08	3.45	5.0	18.4
2	+		90.8		54.49		60.08		106.78		1.96		0.00		26.6
	SEM^d^	0.986	2.600	0.646	1.506	0.338	0.646	2.715	3.449	0.061	0.039	0.725	1.026	3.708	0.000
0		–	85.8^b^	–	51.56^b^	–	60.11	–	100.26	–	1.95	–	0.00	–	22.5
2		–	91.6^a^	–	55.21^a^	–	60.28	–	106.01	–	1.92	–	1.72	–	22.5
	SEM^d^	–	1.839	–	1.065	–	0.457	–	2.439	–	0.028	–	0.726	–	0.000
	–	–	89.8	–	54.21	–	60.36	–	103.21	–	1.91	–	1.72	–	18.4^b^
	+	–	87.6	–	52.57	–	60.03	–	103.06	–	1.96	–	0	–	26.6^a^
	SEM^d^	–	1.839	–	1.065	–	0.457	–	2.439	–	0.028	–	0.726	–	0.000
	VK	0.215	0.046	0.130	0.032	0.381	0.794	0.600	0.121	0.379	0.578	0.316	0.119	0.216	–
*P*-values^3^	SE	–	0.419	–	0.297	–	0.619	–	0.965	–	0.207	–	0.119	–	< 0.010
	VK × SE	–	0.846	–	0.890	–	0.901	–	0.627	–	0.578	–	0.119	–	–

^a, b^Means within a column with no common superscripts differ (*P* < 0.05)

^c^*SE Salmonella* Enteritidis; –, without SE challenge; +, with SE challenge

^d^*SEM* Pooled standard error of the mean

^3^*P*-values for main effect of VK, the main effect of SE challenge, and the interaction between the VK treatments and SE challenge

### Tibia parameters

At week 42, supplementation of VK increased (*P* < 0.05) tibia strength (Table 3). Similarly, at week 44, the main effect of supplementation of VK resulted in higher (*P* < 0.05) tibia strength as well, whereas the main effect of SE challenge resulted in decreased (*P* < 0.05) tibia strength. There was an interaction (*P* < 0.05) between VK and SE on tibia strength. Compared to PS treatment, SE challenged hens fed the VK supplemented diet had a higher (*P* < 0.05) tibia strength. No difference (*P* > 0.05) was observed in medullary area at week 42 (Table 3). At week 44, the main effect of supplementation of VK resulted in lower (*P* < 0.05) medullary area, whereas the main effects of SE challenge increased (*P* < 0.05) medullary area. At week 42, CT was positively correlated with cOC (R = 0.99, *P* = 0.009). Values for PTH and cOC were positively correlated with ucOC (R = 0.96, *P* = 0.036 and R = 0.96, *P* = 0.039, respectively); PTH, OC, and ucOC were negatively correlated with cOC/(cOC + ucOC) (R = − 1.00, *P* = 0.001, R = − 0.95, *P* = 0.046 and R = − 0.96, *P* = 0.039, respectively). At week 44, tibia strength was positively correlated with BMD (R = 0.95, *P* = 0.045), but was inversely related to medullary area (R = − 0.98, *P* = 0.018). Values for PTH were negatively correlated with cOC (R = − 0.97, *P* = 0.035). Those for cOC were positively correlated with cOC/(cOC + ucOC) (R = 0.96, *P* = 0.039), and those for unOC were negatively correlated with cOC/(cOC + ucOC) (R = − 1.00, *P* = 0.004). (Table 4 and 5).

**Table 3:** Effect of dietary vitamin K (VK) supplementation and *Salmonella* Enteritidis (SE) challenge on tibia parameters of laying hens (*n* = 12)

VK, mg/kg	SE^c^	Tibia length, cm		Fresh tibia weight, g		Dry tibia weight, g		Tibia strength, N		Total BMD^d^, g/cm^2^		Medullary area, cm^2^	
		Week 42	Week 44	Week 42	Week 44	Week 42	Week 44	Week 42	Week 44	Week 42	Week 44	Week 42	Week 44
0	–	11.53	11.67	9.73	9.97	4.99	5.13	183.25^b^	235.50^b^	0.29	0.28	1.42	1.59
0	+		11.43		9.39		4.98		209.44^b^		0.28		1.91
2	–	11.66	11.46	9.46	9.61	5.00	5.08	221.14^a^	364.00^a^	0.28	0.32	1.52	0.87
2	+		11.58		9.75		5.25		220.75^b^		0.29		1.65
	SEM^e^	0.079	0.101	0.248	0.253	0.131	0.190	12.320	28.053	0.033	0.023	0.312	0.193
0		–	11.55	–	9.68	–	5.06	–	222.47^b^	–	0.28	–	1.76^a^
2		–	11.53	–	9.69	–	5.17	–	287.60^a^	–	0.30	–	1.26^b^
	SEM^e^	–	0.072	–	0.182	–	0.137	–	20.185	–	0.016	–	0.140
	–	–	11.57	–	9.80	–	5.11	–	295.47^a^	–	0.30	–	1.19^b^
	+	–	11.51	–	9.57	–	5.11	–	215.09^b^	–	0.29	–	1.77^a^
	SEM^e^	–	0.072	–	0.182	–	0.137	–	20.185	–	0.016	–	0.140
	VK	0.260	0.781	0.446	0.984	0.923	0.573	0.049	0.017	0.851	0.306	0.836	0.017
*P*-values^4^	SE	–	0.571	–	0.400	–	0.963	–	0.004	–	0.531	–	0.009
	VE × SE	–	0.094	–	0.168	–	0.418	–	0.045	–	0.589	–	0.253

^a, b^Means within a column with no common superscripts differ (*P* < 0.05)

^c^*SE Salmonella* Enteritidis; −, without SE challenge; +, with SE challenge

^d^*Total BMD* Total bone mineral density

^e^*SEM* Pooled standard error of the mean

^4^*P*-values for main effect of VK, the main effect of SE challenge, and the interaction between the VK treatments and SE challenge

**Table 4:** Pearson’s correlation coefficients between tibia strength, BMD, medullary area and serum biochemical indices at week 42

Week 42	Tibia strength	BMD^a^	Medullary area	Serum Ca^b^	CT^c^	PTH^d^	cOC^e^	ucOC^f^	cOC/(cOC + ucOC)
Tibia strength	1.00								
BMD^a^	−0.43	1.00							
medullary area	0.11	−0.52	1.00						
Serum Ca^b^	−0.63	0.39	−0.19	1.00					
CT^c^	−0.72	0.66	−0.51	0.94	1.00				
PTH^d^	−0.35	0.75	−0.58	0.82	0.89	1.00			
cOC^e^	−0.40	0.55	−0.52	0.93	0.99*	0.94	1.00		
ucOC^f^	−0.42	0.58	−0.38	0.93	0.93	0.96*	0.96*	1.00	
cOC/(cOC + ucOC)	0.38	−0.78	0.61	−0.82	− 0.90	−1.00*	−0.95*	− 0.96*	1.00

^a^*BMD* Bone mineral density

^b^*Serum Ca* Serum calcium

^c^*CT* Calcitonin

^d^*PTH* Parathyroid hormone

^e^*cOC* Carboxylated osteocalcin

^f^*ucOC* Undercarboxylated osteocalcin

*Correlation is significant at *P* < 0.05

Note: The correlation is based on the 6 samples, *n* = 6/treatment, the tibia parameters were the average values of both sides

**Table 5:** Pearson’s correlation coefficients between tibia strength, BMD, medullary area and serum biochemical indices at week 44

Week 44	Tibia strength	BMD^a^	Medullary area	Serum Ca^b^	CT^c^	PTH^d^	cOC^e^	ucOC^f^	cOC/(cOC + ucOC)
Tibia strength	1.00*								
BMD^a^	0.95*	1.00							
medullary area	−0.98	− 0.95	1.00						
Serum Ca^b^	−0.04	0.13	0.17	1.00					
CT^c^	−0.43	−0.23	0.53	0.91	1.00				
PTH^d^	−0.06	−0.31	0.18	−0.03	−0.15	1.00			
cOC^e^	0.29	0.50	−0.42	−0.10	− 0.06	−0.97*	1.00		
ucOC^f^	−0.53	−0.73	0.61	−0.11	− 0.01	0.87	− 0.94	1.00	
cOC/(cOC + ucOC)	0.45	0.67	−0.54	0.11	0.05	−0.91	0.96*	−1.00*	1.00

^a^*BMD* Bone mineral density

^b^*Serum Ca* Serum calcium

^c^*CT* Calcitonin

^d^*PTH* Parathyroid hormone

^e^*cOC* Carboxylated osteocalcin

^f^*ucOC* Undercarboxylated osteocalcin

*correlation is significant at *P* < 0.05

Note: the correlation is based on 6 samples, *n* = 6 per treatment, the tibia parameters were the average values of both sides

### Bone calcium concentration

There were no significant differences (*P* > 0.05) among all treatments with regard to bone calcium indices (Table 6).

**Table 6:** Effect of dietary vitamin K (VK) supplementation and *Salmonella* Enteritidis (SE) challenge on tibia bone calcium (Ca) concentration of laying hens (*n* = 6)

VK, mg/kg	SE^a^	Based on defatted dry matter		Based on total ash	
		Ca^b^, %		Ca^b^, %	
		Week 42	Week 44	Week 42	Week 44
0	–	23.56	21.17	38.74	38.39
0	+		21.86		37.58
2	–	24.36	21.32	40.58	38.82
2	+		21.49		37.08
	SEM^c^	0.739	0.0259	1.021	0.763
0		–	21.51	–	37.98
2		–	21.41	–	37.74
	SEM^c^		0.190	–	0.549
	–	–	21.24	–	38.60
	+	–	21.68	–	37.08
	SEM^c^	–	0.190	–	0.549
	VK	0.472	0.693	0.225	0.688
*P*-values^4^	SE	–	0.114	–	0.055
	VE × SE	–	0.347	–	0.346

^a^*SE Salmonella* Enteritidis; −, without SE challenge; +, with SE challenge

^b^*Ca* Calcium

^c^*SEM* Pooled standard error of the mean

^4^*P*-values for main effect of VK, the main effect of SE challenge, and the interaction between the VK treatments and SE challenge

### Serum biochemical indices

At week 42, there were no differences (*P* > 0.05) in serum biochemical indices between treatments (Table 7). At week 44, the main effect of VK2 treatment revealed higher (*P* < 0.05) CT and cOC levels, as well as cOC/(cOC + ucOC), but lower (*P* < 0.05) ucOC concentrations compared to the VK0 treatment. There were interactions (*P* < 0.05) between VK supplementation and SE challenge for Ca. However, at week 44, hens without supplemental VK had a higher (*P* < 0.05) serum Ca content compared to the PS treatment.

**Table 7:** Effect of dietary vitamin K (VK) supplementation and *Salmonella* Enteritidis (SE) challenge on serum biochemical indices of laying hens (*n* = 6)

VK, mg/kg	SE^c^	Ca^d^, mmol/L		CT^e^, pg/mL		PTH^f^, pg/mL		cOC^g^, ng/mL		ucOC^h^, ng/mL		cOC/(cOC + ucOC), %	
		Week 42	Week 44	Week 42	Week 44	Week 42	Week 44	Week 42	Week 44	Week 42	Week 44	Week 42	Week 44
0	–	5.83	3.47^b^	321.53	231.04	102.19	98.26	2.38	2.07	2.10	2.43	57.92	44.33
0	+		5.40^a^		330.98		99.39		1.87		2.46		43.90
2	–	5.03	4.66^ab^	270.89	262.15	67.43	92.20	2.32	2.45	1.54	1.45	59.75	63.70
2	+		4.57^ab^		301.62		81.93		2.76		1.39		67.64
	SEM^i^	0.973	0.406	29.193	25.980	15.255	8.219	0.301	0.100	0.400	0.285	0.731	2.539
0		–	4.37	–	246.60^b^	–	98.64	–	1.99^b^	–	2.44^a^	–	44.16^b^
2		–	4.62	–	314.20^a^	–	88.78	–	2.52^a^	–	1.43^b^	–	64.69^a^
	SEM^i^	–	0.287	–	17.770	–	5.897	–	0.073	–	0.179	–	2.273
	–	–	4.06^b^	–	281.01	–	95.23	–	2.67	–	2.01	–	54.02
	+	–	4.98^a^	–	284.71	–	90.66	–	2.76	–	1.92	–	51.81
	SEM^i^	–	0.287	–	17.770	–	5.811	–	0.071	–	0.178	–	2.244
	VK	0.622	0.661	0.345	0.022	0.248	0.20	0.898	< 0.001	0.424	0.005	0.220	0.001
*P*-values^8^	SE	–	0.032	–	0.973	–	0.60	–	0.662	–	0.959	–	0.611
	VK × SE	–	0.019	–	0.260	–	0.513	–	0.051	–	0.861	–	0.530

^a, b^Means within a column with no common superscripts differ (*P* < 0.05)

^c^*SE Salmonella* Enteritidis; −, without SE challenge; +, with SE challenge

^d^*Ca* Calcium

^e^*CT* calcitonin

^f^*PTH* Parathyroid hormone

^g^*cOC* Carboxylated osteocalcin

^h^*ucOC* Undercarboxylated osteocalcin

^i^*SEM* Pooled standard error of the mean

^8^*P*-values for main effect of VK, the main effect of SE challenge, and the interaction between the VK treatments and SE challenge

## Discussion

According to the results of the present study, dietary supplementation of 2 mg/kg VK maintained higher laying rate, egg mass, and tibial strength of hens at week 42 of age.

There are no reports so far on the effect of supplemental VK on performance of laying hens challenged with SE, but similar to our findings in non-challenged birds [18] did not observe any effect of 10 mg/kg VK addition to the diet of LSL White Leghorn laying hens on birds’ egg production. If birds were challenged with SE, however, a higher mortality rate was obtained in the present study which is in agreement with observations in Arbor Acre broilers [5].

Bones as dynamic organs are constantly remodelled by osteoclasts and osteoblasts [19], and VK plays an important role in bone development and quality [20]. Thus, long-term VK deficiency may result in the loss of bone mass [21]. In the present study, VK supplementation increased the tibia strength at week 42. This is in agreement with results of a study by Guo [22], where dietary supplementation of 0.5 and 4.0 mg/kg of VK increased tibia breaking strength of male broilers on d 21. Following SE challenge, lower tibia strength points towards the decrease of BMD [23]. Similarly, Higgins et al. [24] and Ikejiri K et al. [25] reported bone deformity and bone infections, respectively, when birds were challenged with SE, and humans were infected with SE. In the present study, the main effect of supplementation of VK resulted in increased tibia strength, however, the main effect of SE challenge resulted in decreased tibia strength. This finding is in support of previous work, where dietary VK has shown not only to improve bone formation, but also to inhibit bone degradation [8]. Furthermore, tibia strength was positively correlated with BMD (R = 0.95, *P* = 0.045) and negatively correlated with medullary area (R = − 0.98, *P* = 0.018) at week 44, which is in support of the observation that tibia strength might be influenced by minerals [2]. However, it remains open, why supplemental VK improved tibia strength of non-challenged birds, but did not alleviate tibia strength upon SE challenge. More studies are needed to address the relationship between dietary VK and tibia strength under SE challenge conditions.

Homeostasis of Ca is an important driving force in the maintenance of bone strength as Ca is released into the blood stream during bone resorption and deposited into bones during bone formation [11]. In the present study, SE challenge increased serum levels of Ca suggesting SE may induce a defect in Ca homeostasis. The serum Ca content of challenged birds was similar to levels determined in non-challenged birds fed a diet supplemented with 2 mg/kg VK, however, it was higher than in non-challenged birds fed a diet devoid of supplemental VK. This phenomenon points towards the release of Ca from the bone into the blood. In addition, VK serves as a cofactor of endoplasmic reticulum resident γ-glutamyl carboxylase (GGCX), which carboxylates any selected glutamate residues on the target proteins and enables these proteins such as cOC to bind to Ca. Due to this function, VK may increase the affinity of cOC for Ca ions and hydroxyapatite crystals, and hence facilitate bone formation [26].

Calcitonin (CT) inhibits bone resorption and stimulates bone growth by rapidly lowering serum Ca levels and decreasing Ca released from bone, respectively [27–29]. In the present study, supplementation of 2 mg/kg VK to the diet of SE challenged birds increased serum CT levels, indicating that VK might be able to ameliorate SE-induced bone damage as well as maintain serum Ca balance.

Osteoblasts present in bones are known to synthesize cOC as precursor, which is then γ-carboxylated in the presence of VK, followed by incorporation into bone matrix, where cOC is involved in bone calcification. In the present study, VK supplementation increased the cOC levels in serum in comparison to the treatment devoid of supplemental VK in the diet. It appears that VK has a beneficial effect on bone formation, thus contributing to improved tibia strength. Previously, cOC has been described as a marker of bone formation [30], and as the most sensitive known serum marker for VK status in bone tissue [31]. In addition, OC has 3 γ-carboxyglutamic acid residues [32], and can bind with Ca and hydroxyapatite. In addition, cOC regulates the rate of mineral maturation through expanding hydroxyapatite crystal size [33]. Therefore, it is assumed that cOC plays a vital role in skeletal development, and is considered as marker of bone formation [34–36]. As coenzyme for γ-carboxylase [37], VK plays an important role in bone metabolism as well [38].

During deficiency of VK, OC does not undergo complete γ-carboxylation, and ucOC is released from osteoblasts into the circulating blood [39]. In this context, ucOC has been described as potential risk factor for bone fracture [10, 35]. Therefore, serum levels of ucOC may reflect birds’ nutritional status with regard to VK intake. Moreover, ucOC appears to be a negative regulator of bone formation [40]. These findings are in support of the present results, where VK supplementation decreased serum levels of ucOC and increased the affinity of Ca to the bone matrix [41] which, in turn, contributed to increased tibia strength. Consequently, the greater tibia strength due to VK supplementation might reflect improved balance between bone formation markers such as cOC and fracture risk factors such as ucOC.

The skeleton comprises compact bone and medullary bones [42], but infection with SE may initiate bone resorption from the medullary bones only, without affecting the compact bones. As a result, osteoblastic bone formation can be impaired and bone resorption will be enhanced, thereby contributing to increased bone fragility and fracture risk. According to the World Health Organization (WHO), porous bones in association with deteriorated bone tissue result in low BMD values, also referred to as osteoporosis. In this study, supplementation of VK decreased in SE-challenged birds the medullary area due to beneficial effects of VK on bone formation. Without dietary VK supplementation, however, SE challenge caused an increase of medullary area as bone resorption was enhanced.

## Conclusions

Dietary supplementation of 2 mg/kg VK may mitigate the Ca homeostasis disruptions caused by SE challenge, and can be used as dietary intervention for control of *Salmonella* infection in laying hens through strengthening of birds’ skeleton. Medullary area has proven to be a sensitive biomarker for bone calcium loss caused by SE infection. Further knowledge of the various metabolic functions of VK may aid in developing novel feeding strategies to secure both performance and health of laying hens.

## Data Availability

Not applicable.

## Abbreviations

BMD: Bone mineral density
Ca: Calcium
cOC: Carboxylated osteocalcin
CT: Calcitonin
ELISA: Enzyme-linked immunosorbent assays
OC: Osteocalcin
PBS: Phosphate buffer saline
PS: Physiological saline solution
PTH: Parathyroid hormone
SE: *Salmonella* Enteritidis
ucOC: Undercarboxylated osteocalcin
VK: Vitamin K
XLD: Xylose lysine doxycholate
